# 4403 Cure Quest: Team Science of Game Design for Medical Education

**DOI:** 10.1017/cts.2020.205

**Published:** 2020-07-29

**Authors:** Benjamin Chang, Shawn Lawson, Kathleen Ruiz, Mei Si, Emilia Bagiella, Emma K T Benn, Janice Lynn Gabrilove

**Affiliations:** 1 RPI, School of Humanities, Arts and Social Sciences; 2 Rensselaer Polytechnic Institute; 3 Mount Sinai School of Medicine

## Abstract

OBJECTIVES/GOALS: “Cure Quest” is an adventure quest game about the process of new drug discovery and development. The player explores a magical island in search of a cure for a mysterious illness, traveling through different lands based on the stages of the drug discovery pipeline. Along the way, they must solve puzzles, decipher clues, and enlist the help of a ‘team science’ group of collaborators. The game uses a fantastical setting and engaging story to communicate the topic through metaphorical representations, instilling a sense of wonderment in the learning process. Real-world science is embedded into fictionalized lands such as the Labyrinth of Target Identification, the Forest of Small Molecule Discovery, the Tree of Biostatistics, the Mountains of FDA Approval and the Desert of Funding. The project represents a novel application of game-based learning to a complex topic not typically addressed through games. The process of designing and developing the game itself uncovers strong parallels between the interdisciplinary game design process and the interdisciplinary team science process. The objective of the game is to communicate high-level concepts of the drug discovery and development process, starting with the principles of ethical research and the motivations behind medical discovery, through the development of a new drug and finally to FDA approval. The goal is to improve understanding of clinical translational science among the different disciplines involved, and to raise overall awareness of the drug discovery process. METHODS/STUDY POPULATION: The game is being developed through a collaboration between faculty and students at ISMMS and the Games and Simulation Arts and Science Program at Rensselaer Polytechnic Institute. The first target audience is 2nd-3rd year medical students, with the future goal of adapting the game to a broader population. The game design is informed by specific learning outcomes, input from players in the target population and an ongoing iterative design process. The game is designed for mobile devices (iOS and Android), with an emphasis on narrative, exploration, and puzzle solving. Future evaluation will be performed through a quasi-experimental design comparing standard lectures with the game on a drug discovery. RESULTS/ANTICIPATED RESULTS: The game is currently in development, but the project has yielded insight into the design process for serious games in medicine. We found that for a game of this type it is essential not just to have both designers and subject matter experts, but to enable cross-pollination of modes of thinking. Through multiple design iterations and focus groups, we found that a game design approach rooted in narrative and allegorical abstraction would have a better ability to engage the target audience than one focused only on realistic simulation. When complete, we anticipate that the game will improve understanding of the core concepts in drug discovery. CureQuest is designed as an episodic game, following the sequence of stages in the drug discovery and development process. In this version of the game, we demonstrate five of the initial episodes: The City of Discovery of Unmet Medical Need; The Labyrinth of Target Identification; the Aquarium of Transgenic Phenotype Expression; the Rival Researcher Gang Quiz Battle; and the Desert of Funding. DISCUSSION/SIGNIFICANCE OF IMPACT: If successful, the game-based learning approach can help fill key gaps in current formal medical and scientific training, as well as gaps in understanding among the general public. The design process serves as an informative model of evolving collaborative team science.

